# Slit dual-frequency ultrasound-assisted pulping of *Lycium barbarum* fresh fruit to improve the dissolution of polysaccharides and in situ real-time monitoring

**DOI:** 10.1016/j.ultsonch.2023.106509

**Published:** 2023-07-01

**Authors:** Tianyu Kong, Shuhan Liu, Yuqin Feng, Yanli Fan, Junwei Yu, Haihui Zhang, Meihong Cai, Haile Ma, Yuqing Duan

**Affiliations:** a School of Food and Biological Engineering, Jiangsu University, Zhenjiang 212013, China; b School of Food & Wine, Ningxia University, Yinchuan 750021, China; c Ningxia Zhongning Goji Industry Innovation Research Institute Co., Ltd, Zhongning 755100, China; d Institute of Food Physical Processing, Jiangsu University, Zhenjiang 212013, China; e Nourse Pet Nutrition Jiangsu Research Institute, Zhenjiang 212009, China

**Keywords:** *Lycium barbarum* pulp, Polysaccharides, Dissolution kinetic model, Structural properties, Antioxidant, In situ real-time monitoring

## Abstract

In this study, the slit dual-frequency ultrasound-assisted pulping of fresh *Lycium barbarum* fruit was optimized to improve the dissolution of polysaccharides. The microscopic mechanism of polysaccharide dissolution was explored through establishing polysaccharides dissolution kinetics model and visualizing the multi-physical fields during ultrasonic process, and an in situ real-time monitoring model was established by the relationship between the chemical value and spectral information collected by near-infrared spectroscopy. The results showed that, under optimal conditions, treatment with ultrasound (28–33 kHz, 250 W, 30 min) not only significantly promoted the dissolution rate of polysaccharides in *Lycium barbarum* pulp (LBPPs, increased by 43.64 %, *p* < 0.01), reduced its molecular weight, but also improved the arabinose molar ratio, the uniformity of polysaccharide particles, and the antioxidant activity of LBPPs. Correlation analysis indicated that ultrasonic treatment is closely related to LBPPs content, particle size and scavenging capacity against superoxide anion radicals (*p*_total sugar content_ < 0.01, *p*_particle size_ < 0.05 and *p*_superoxide anion scavenging_ < 0.05). Moreover, the in situ real-time monitoring model for the pulping process could quantitatively predict LBPPs dissolution rate and its superoxide anion radical scavenging capacity with good calibration and prediction performance (*Rc* = 0.9841, *RMSECV* = 0.0873, *Rp* = 0.9772, *RMSEP* = 0.0530; *Rc* = 0.9874, *RMSECV* = 0.1246, *Rp* = 0.9868, *RMSEP* = 0.0665). These results indicated that slit dual-frequency ultrasound has great potential in improving the quality of *Lycium barbarum* pulp, which may provide theoretical support for the industrial development of intelligent systems for polysaccharides preparation.

## Highlights

• Slit dual-frequency ultrasound-assisted pulping of *Lycium barbarum* was optimized.
• A model for dissolution kinetics of *Lycium barbarum* polysaccharides was developed.
• The visualization of multiple-physical fields in ultrasonic process was studied.
• *Lycium barbarum* polysaccharide dissolution and antioxidant activity were enhanced.
• An in situ real-time monitoring model of *Lycium barbarum* pulping was established.

## Graphical abstract

**Figure:**
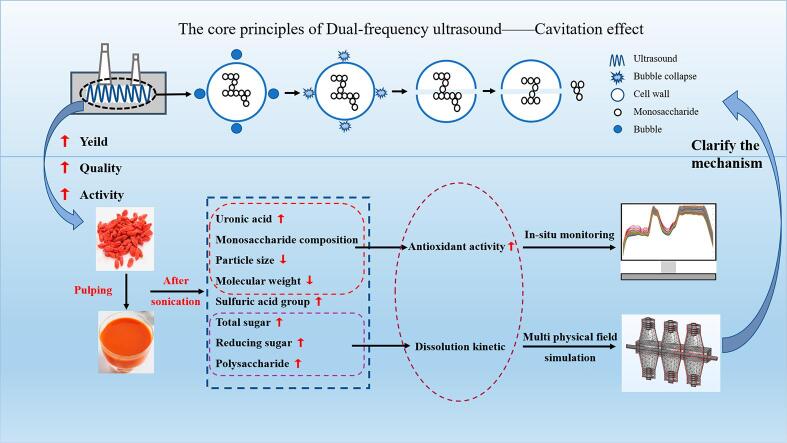

## 1 Introduction

*Lycium barbarum* is the berry of perennial shrubs distributed in most parts of China, Europe, and the Mediterranean region, it has been used as homologous medicine and food in China for thousands of years. *Lycium barbarum* is rich in polysaccharides, alkaloids, flavonoids, polyphenols, and amino acids [1]. Among various constituents, *Lycium barbarum* polysaccharides with glycan-O-Ser glycopeptide structure are considered the most important bioactive components [2], [3] with anti-tumor [4], anti-inflammatory, hypoglycemic and hypolipidemic activities, especially antioxidant effects [5]. Therefore, the effective content of polysaccharides has become an important indicator to measure the quality of *Lycium barbarum* products.

*Lycium barbarum* is mainly used in the dried form, or as freshly squeezed fruit juice. Compared with dry fruit products, *Lycium barbarum* pulp (LBP) is favored by consumers due to its high nutrient retention rate. Traditional LBP pulping process often fails to fully utilize the nutrient in the peel and seeds, leading to significant waste of raw materials, and high energy consumption. Treatment with hot water (80–100 °C) for 2 h or more is beneficial to the dissolution of polysaccharides [6]. However, due to the low efficiency, time-and energy consuming of heat treatment [7] and the loss of heat-sensitive nutritional ingredients, it is not conducive to the preparation of LBP with fresh fruit. Current market calls for more convenient and high-quality LBP products with high nutrient retention rate. In view of this, the innovation of pulping technology has become an urgent demand of *Lycium barbarum* industry.

In recent years, some novel physical processing techniques, such as ultrasound [8], [9], microwave [10], pulsed electric field [11], ohmic heating [12], radio frequency [13], and their combinations [14], [15], [16], have been used to facilitate the dissolution of polysaccharides from various food matrices, showing obvious advantages over traditional methods in many aspects. Among them, low-frequency and high-intensity ultrasonic treatment is the most cost-effective and feasible application in polysaccharide processing [17]. Under this mode, ultrasonic cavitation generates strong shear force and turbulence [18], and the cell wall is destroyed by acoustic energy, forming microchannels inside the material, promoting mass transfer and solvent penetration [19], which is the main driving force for improving polysaccharide extraction [20], In addition, ultrasonic assisted processing can improve the physical properties of food (dry matter content and color), thus affecting consumers' choice and reducing the loss of products [21], [22]. Moreover, ultrasonic degradation enhances the biological activity of polysaccharides by altering their structure, such as molecular weight reduction and hydrogen bond breakage [23], [24]. Notably, ultrasonic frequency modes have great influence on cavitation intensity. The number of bubbles generated by multi-frequency ultrasound is five times that of single-frequency ultrasound, thus resulting in much stronger cavitation effect [25], [26]. Studies show that dual frequency ultrasound can effectively overcome cavitation shielding [27], avoid the problems of large standing wave area and uneven sound field existing in single frequency ultrasound [28], [29], so as to significantly improve the yield of plysaccharides from *Lycium barbarum* [23], *Lentinus edodes* [30] and *Fructus aurantii* [31], and enhance their physicochemical properties, antioxidant, anti-tumor and immune activities, which is better than that of single/triple frequency ultrasound and hot water treatment. However, the specific situation of the physical field in the ultrasonic process needs to be verified by finite element coupling simulation [32], [33], [34]. Based on this, we speculated that the combination of dual-frequency ultrasonic treatment and traditional pulping may be an effective means to promote the dissolution of polysaccharides and the other nutrients, thereby improving the nutritional value of LBP.

Kinetic models can facilitate the design, control and optimization of the experimental processes, as well as identify the trend of changes in target ingredients under different conditions. At present, diffusion model, pseudo first order model, and two-point kinetic models are commonly used to simulate and analyze the solid–liquid extraction process of polysaccharides [35], which can precisely control the process variables and provide useful information for future industrial applications [36]. However, up to now, no kinetic model related to polysaccharides dissolution during ultrasonic assisted pulping of *Lycium barbarum* has been reported. In addition, the monitoring of target components in the current extraction process mainly depends on traditional offline physical and chemical detection, which often requires long time and cannot monitor the dynamic changes of target components in real time [37]. In addition, the use of organic reagents may cause damage to the sample. Compared with traditional monitoring technologies, near-infrared (NIR) spectral monitoring technology is fast, economic, objective, accurate and nondestructive [38], it has rapidly developed into a reliable analysis method for detecting the molecular structure information of hydrogen containing organic compounds [39]. Currently, NIR spectroscopy monitoring technology has made breakthroughs in many fields of the food industry, such as monitoring the content changes of polysaccharides [40], [41], proteins [42] and vitamins [43] in food, controlling and evaluating food quality and safety [44], accurately identifying food sources [45], and so on. With the continuous updates of micro NIR fiber probes and spectral systems, it has become more convenient to place it in experimental containers for real-time monitoring of polysaccharides and activity. However, due to significant differences in near infrared absorption wavelengths and intensities between different groups (such as methyl, methylene, benzene ring) or the same group in different chemical environments, the monitoring of relevant indicators in the process of *Lycium barbarum* pulping is currently insufficient.

Therefore, in order to improve the dissolution of polysaccharides in LBP (LBPPs) and the other nutrients, we optimized the slit dual-frequency ultrasound-assisted pulping of *Lycium barbarum* fresh fruit, constructed a model of polysaccharide dissolution kinetics in this study, and the effects of ultrasonic treatment on monosaccharide composition, molecular weight, particle size and antioxidant activity of LBPPs were also investigated. In addition, an in situ real-time monitoring model was established according to LBPPs dissolution rate and spectral information collected by NIR spectroscopy, which may provide a theoretical support for the intelligent control of polysaccharide dissolution during slit dual-frequency ultrasound assisted fresh fruit pulping.

## 2 Materials and methods

### 2.1 Materials and chemicals

Fresh fruits of *L. barbarum* (provided by Zhongning, Ningxia, China). Polysaccharide pellet radius (R), calculated approximately 1 mm. Ethanol, sulphuric acid, phenyl hydroxide, methenyl chloride, normal butanol, sodium hydroxide (NaOH), Folin-Ciocalteu reagent, arabinose, D-glucose, xylan, mannose, galactose, rhamnose, 2, 2′-azinobis (3-ethylbenzothiazoline-6-sulphonic acid) (ABTS^+^•), 1,1-Diphenyl-2-picrylhydrazyl radical 2,2-Diphenyl-1-(2,4,6-trinitrophenyl) hydrazyl (DPPH•), methanol, and pyridine were purchased from Sinopharm Chemical Reagent Co., Ltd. (Shanghai, China), phenazine methosulfate (PMS), nitro blue tetrazolium salt (NBT), dihydronicotineamidadenine dinucleotide (NADH).

### 2.2 Equipment

The main devices applied in this study were as follows: Micro ultraviolet spectrophotometer (UV–Vis), Fourier infrared spectrometer (FT-IR), Gas chromatograph (7890 A, Agilent Technologies, PaloAlto, CA, USA), Multi angle laser scattering detector (SEC-MALLS, DAWN HELEOS II, Wyatt Technology Co., Santa Barbara, CA, USA), Laser light scattering instrument (Anton Paar Litesizer 500, Graz, Austri).

**Fig. 1:**
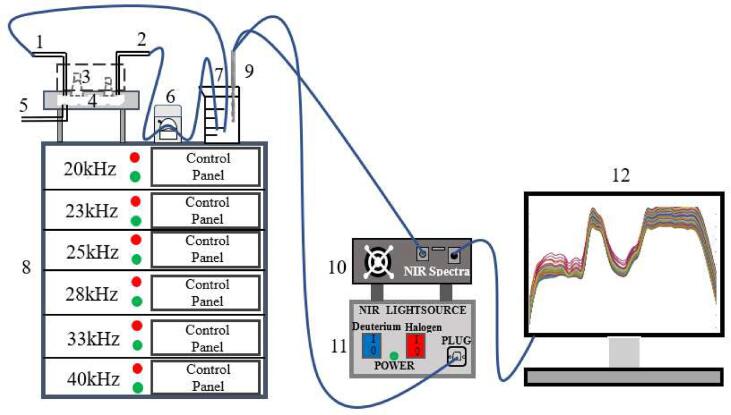
Ultrasound equipment and in-situ real-time monitoring system. (1) Exit port; (2) Injection port; (3) Ultrasound transducer; (4) Slit cavity; (5) Contaminants export; (6) peristaltic pump; (7) LBP; (8) Ultrasonic generator; (9) Micro fiber optic probe; (10) Near-infrared spectroscopy; (11) Light source; (12) Data analysis platform.

The six-frequency slit divergent ultrasound equipment (Fig. 1) developed by Jiangsu University is composed of transducer, ultrasonic generator, slit chamber, PLC (programmable logic controller) control system and condensate circulating system [46]. This equipment can work under different frequencies (20, 23, 25, 28, 33 and 40 kHz) and their combinations, it has a processing chamber with divergence and convergence angles, which can significantly reduce the standing wave effect, and increasing the contact frequency between ultrasonic field and materials, thereby solving the problem of uneven energy distribution of concentrated and divergent ultrasound. The sample can be circulated into the ultrasonic slit chamber through a peristaltic pump for processing.

Micro fiber NIR spectrometer (NIRQUEST256-2.5, Ocean Optics, Largo, FL, USA) consists of a fiber optical probe (TP300 immersion optical fiber probe, Ocean Optics, American) and a portable halogen light source (DH-2000-BAL,Ocean Optics, American) (Fig. 1). The spectral information was collected by completely immersing the optical fiber probe into liquid, and the changes of related indices were monitored in real-time throughout the pulping process.

In the process of ultrasound-assisted pulping, LBP samples were collected every 20 s, and the integration time was set as 200 ms for three consecutive scans to generate NIR spectra. The scanning spectral region of NIR spectrometer was 800–2500 nm. A total of 256 spectra were acquired with a fiber-optic probe (resolution: 6.4 cm^−1^; sliding width: 2). Each raw spectrum was the average of 3 scanned spectra. Under approximate experimental conditions, the background spectra were collected using distilled water before each NIR measurement.

### 2.3 Fresh fruit pulping of *Lycium barbarum*

Before ultrasonic treatment, the *Lycium barbarum* fresh fruit was cleaned and then crushed by a beating machine to make LBP for further use [47]. Based on previous literature [48], ultrasonic power, treatment time, and synchronous dual-frequency modes were selected to carry out single-factor experiments. At room temperature, LBP samples were fed into the ultrasonic equipment through a peristaltic pump. The ultrasonic conditions were as follows: power was respectively 150 W, 200 W, 250 W, 300 W, 350 W; sonication time was respectively 10, 20, 30, 40, 50 min; dual-frequency combination were respectively 23–28 kHz, 25–28 kHz, 28–33 kHz, and 28–40 kHz. The optimal level of each factor was preliminarily determined by single-factor experiments.

### 2.4 Response surface methodology (RSM)

Based on single factor experiments, the response surface methodology was used to further optimize the pulping process conditions (*X_1_*, ultrasonic power; *X_2_*, ultrasonic dual- frequency combination; *X_3_*, ultrasonic time). The whole design was composed of 17 runs, and coded variables appearing at three levels (−1, 0, and 1) according to the following Equation (1) [49]:

(1) $$x_{i}=\frac{X_{i}-X_{0}}{\Delta X}$$

where *X_i_* is a coded value of variable; *X_i_* is the actual value of variable; *X_0_* is the actual value of *X_i_* on the center point; and *ΔX* is the step change value.

As shown in Table 1, five replicates of the center (13–17) were designed to estimate the variability and stability [50]. All tests were performed in triplicate. Taking dissolution rate as the response value, statistical analysis and the establishment of regression model were carried out by Design Expert 10 software.

**Table 1:** Response design matrix and response values for ultrasound-assisted LBP pulping process.

Number	X_1_	X_2_	X_3_	Dissolution rate (%)
1	1 (300 W)	1 (28–33 kHz)	0	6.29 ± 0.09
2	1	−1 (23–28 kHz)	0	6.36 ± 0.12
3	0 (250 W)	1	1 (40 min)	6.41 ± 0.24
4	1	0 (25–28 kHz)	1	6.46 ± 0.10
5	−1(200 W)	0	−1 (20 min)	6.50 ± 0.22
6	−1	0	1	6.52 ± 0.14
7	−1	1	0	6.57 ± 0.13
8	0	−1	−1	6.62 ± 0.21
9	−1	−1	0	6.62 ± 0.12
10	0	−1	1	6.68 ± 0.11
11	0	1	−1	6.69 ± 0.16
12	1	0	−1	6.80 ± 0.09
13	0	0	0	7.44 ± 0.20
14	0	0	0	7.50 ± 0.14
15	0	0	0	7.52 ± 0.21
16	0	0	0	7.56 ± 0.33
17	0	0	0	7.57 ± 0.17

X_1_, Ultrasonic power (W); X_2_, Ultrasonic frequency (kHz); X_3_, Ultrasonic Time (min).

The multiple regression analysis of RSM conforms to the following quadratic polynomial model.

(2) $$y_{k}=b_{\mathrm{ko}}+\sum_{i=1}^{3}b_{k_{i}}x_{i}+\sum_{i=1}^{3}b_{\mathrm{kii}}x_{i}^{2}+\sum_{i<j=2}^{3}b_{\mathrm{kii}}x_{i}x_{j}$$

*y_k_* is the response function; *b_k0_*, *b_ki_*, *b_kii_*, and *b_kij_* represent the regression coefficients for intercept, linear, quadratic and interaction terms, respectively; *x_i_* and *x_j_* are the independent variables. Additional confirmation experiments were subsequently conducted to verify the validity of the experimental design.

### 2.5 Pretreatment of LBP

Obtained LBP were centrifuged at 4000 rpm / min for 15 min, the supernatant was collected and concentrated to 1/5–1/4 of the original volume with a rotary evaporator at 60 °C, then added 4 volumes of absolute ethanol, refrigerated at 4 °C overnight, centrifuged at 4000 rpm for 15 min, and collected the sediment. The protein in the sediment was removed by Sevage method (trichloromethane: n-butanol 4:1, v/v) [51]. The retained solution was dialyzed and then freeze-dried to obtain crude LBPPs (non-ultrasonic treatment: NU-LBPPs; ultrasonic treatment: U-LBPPs) for further analysis.

### 2.6 Determination of main nutrients in LBP and LBPPs

The content of total sugar was determined by phenol sulfuric acid method with D-glucose as the standard [52]. Reducing sugar was measured by DNS colorimetry [53], and the content of polysaccharide was determined by subtracting the reducing sugar content. Polyphenol and protein were respectively determined by Folin-phenol method [54] and Coomassie brilliant blue method [55]. The content of uronic acid was tested by m-hydroxybiphenyl method using D-galactoglucuronic acid as the standard [56]. The determination of carotenoid was based on the improved organic solvent extraction method [57]. The content of sulfate was determined by gelatin barium chloride method [58]. The contents of different components in LBP and LBPPs before and after ultrasonic treatment were determined in detail, and further comparative analysis was performed.

### 2.7 Dissolution kinetics of LBPPs in ultrasound-assisted pulping process

The LBP was sonicated under different sonication power (150 W, 200 W, 250 W, 300 W) and dual-frequency combinations (23–28 kHz, 25–28 kHz, 28–33 kHz, 28–40 kHz), and the contents of total sugar and reducing sugar in LBP were determined by sampling at 5, 10, 15, 20, 25, 30, 35, 40, 45, 50 min. Then the dissolution rate of LBPPs was determined for further analysis of model parameters.

The dissolution of LBPPs is a mass transfer process, usually divided into three steps:

a) During beating process, water is mixed with LBP to dissolve polysaccharides;
b) Dissolved polysaccharides diffuse from the inner surface of LBP particles to the outer surface;
c) Polysaccharides diffuse from the outer surface of LBP particles to the solution [59].

The second stage is a non-steady state diffusion process that requires more time [60], therefore the osmotic diffusion of solvents in LBP is the controlling step of LBPPs dissolution. In this study, LBP was considered to be composed of uniform spherical particles with a radius of r, and the following assumptions were made for model building:

a. The *Lycium barbarum* granules are approximately spherical particles with uniform size from pulping to the end, and their shape remains almost unchanged.
b. The mass transfer resistance at the particle surface can be neglected [61].
c. Polysaccharides only diffuse along the inner diameter and have a homogeneous mass concentration of diffusion.
d. The diffusion coefficient of polysaccharides remains constant at a constant dissolution temperature.

Based on the above assumptions, the globular kinetic model equation of Fick's second law can be expressed as [62]:

(3) $$\frac{\partial C}{\partial t}=D_{S}\left(\frac{\partial^{2}C}{\partial t^{2}}+\frac{2}{r}\right)\frac{\partial C}{\partial t}$$

where *C* is the polysaccharide concentration (mg/mL) of LBP; *r* is the distance from the surface to the center (mm); *Ds* is the effective diffusion coefficient of polysaccharides (mm^2^/s); and *t* is the ultrasound time (min).

The Eq. (3) could be simplified as Eq. (4):

(4) $$\frac{\partial C}{\partial t}=D_{S}\frac{\partial^{2}C}{\partial r^{2}}$$

The initial and boundary conditions are described as follows:

$$t=0,C=C_{0},r=0;r=R,V\cdot \frac{\partial C}{\partial t}=-D_{S}\cdot S\left(\frac{\partial C}{\partial r}\right)r=R,$$

where *C_0_* is the mass concentration (mg/L) of polysaccharides in LBP at the initial time; *V* is the volume of solvent; *S* is the contact area between LBP particles and solvent. Eq. (5) is obtained by solving the ordinary differential equation through Fourier transform [63].

(5) $$\frac{{(C}_{\infty }-C)}{{(C}_{\infty }-C_{0})}=\left(\frac{6}{\pi^{2}}\right)\sum_{n=1}^{\infty }\left\{\left[\frac{1}{n^{2}}\right]exp\left[-{\left(\frac{n\pi }{R}\right)}^{2}D_{s}t\right]\right\}$$

where *C∞* is the mass concentration of polysaccharides in LBP when the dissolution reaches equilibrium. Due to the negligible convergence of the high-order term of concentration to zero, when *n* = 1 [64], *C_0_* = 0, the logarithm on both sides of Eq. (5) can be taken to obtain Eq. (6) [65].

(6) $$Ln\left[\frac{C_{\infty }}{\left(C_{\infty }-C)\right]}\right]=kt+b$$

(7) $$k=\frac{\pi^{2}D_{s}}{R^{2}}$$

where *k* denotes the extraction rate constant (s^−1^), *b* is the intercept. The relative raffinate rate (*y*) is described as follows:

(8) Relativeraffinaterate:y=C∞-CC∞=a·exp-kt

(9) $$Half-life\;period:t_{\frac{1}{2}}=\frac{\left[Ln2-Ln\left(\frac{\pi^{2}}{6}\right)\right]}{k}$$

### 2.8 Multi physical field coupling simulation of slit ultrasound

In order to further study the ultrasonic field in the slit ultrasonic reaction chamber, the visualization effect of the distribution of sound field, flow field and temperature field were realized through the finite element simulation software COMSOL Multiphysics 5.6. According to previous studies, the distributions of sound field, flow field and temperature field were constructed and analyzed by the theory of piezoelectric effect [32], [66], [67], acoustic flow effect [67], [68] and ultrasonic thermal effect [69], [70].

### 2.9 Structural characteristics of LBPPs

#### 2.9.1 UV–Visible (UV–Vis) spectroscopy analysis

The ultraviolet absorbance of various LBPPs solution (1 mg/mL) in the range of 190–400 nm was measured by an ultraviolet/visible spectrophotometer (Cali-100, Varian, Palo Alto, California, USA).

#### 2.9.2 FT-IR spectroscopy analysis

NU-LBPPs and U-LBPPs samples (1–2 mg) were respectively milled with 100–200 mg KBr (1:100) to form 1 mm particles for FT-IR analysis (Nicolet iS50 FT-IR Spectrometer, Thermo Electron, Madison, WI, USA), in a frequency range of 4000–400 cm^−1^.

#### 2.9.3 Monosaccharide composition analysis

The monosaccharide composition of LBPPs was determined according to a reported method with minor modification [9]. Briefly, 10 mg of LBPPs and 4 mL of 2 mol/L trifluoroacetic acid were put into an ampoule and reacted at 110 °C for 8 h, followed by neutralization with BaCO_3_ and centrifugation at 5000 rpm for 10 min, then the supernatant was concentrated to obtain the polysaccharide hydrolysates under reduced pressure.

The polysaccharide hydrolysates reacted with 1 mL pyridine, 10 mg hydroxylamine hydrochloride and 1 mg internal standard myoinositol at 90 °C for 0.5 h, then 1 mL acetic anhydride was added to acetylate for 0.5 h. After cooling, the polysaccharide hydrolysates were filtrated with 0.22 μm organic phase filter membrane. Each monosaccharide standard was derivatized as the procedure described above [71]. The derivative was analyzed by gas chromatograph.

#### 2.9.4 Analysis of glycan-peptide linkage and side chain branching

The carbohydrate-peptide linkage of LBPPs and the branches and side chains were respectively analyzed by *β*-Elimination reaction [72] and I_2_-KI reaction [73], the samples were scanned from 200 to 400 nm by UV-Vis Spectrum.

#### 2.9.5 Molecular weight analysis

The relative molecular weight (*Mw*) of LBPPs was determined by a previous method with slight modification [71]. Briefly, the weight-average (*Mw*), number-average molecular weight (*Mn*), molecular weight distribution (*Mw*/*Mn*) of LBPPs were determined by size-exclusion chromatography coupled with multiangle laser light scattering. OHpak SB-806 MHQ and SB-805 HQ gel chromatographic columns were connected in series, and OHpak SB-G was used as a guard column; the column temperature was 25 °C, the flow rate was 0.5 mL/min, the sample injection volume was 200 µL, and the refractive index increment was 0.138 mL/g.

#### 2.9.6 Determination of particle size

The size distribution of NU-LBPPs and U-LBPPs (0.02 mg/mL) was determined by laser scattering instrument under static light scattering conditions. The measurement was carried at room temperature, and the laser wavelength was 532 nm.

### 2.10 Evaluation of *in vitro* antioxidant activity

#### 2.10.1 DPPH• radical scavenging activity

DPPH• scavenging activity assay was performed according to a modified method [74]. In brief, 1 mL of LBPPs (0.1, 0.2, 0.4, 0.6, 0.8, 1 mg/mL) was incubated with 1 mL of DPPH• ethanol solution (0.2 mmol) at room temperature in the dark for 30 min, and the absorbance value was measured at 517 nm with a microplate reader.

#### 2.10.2 ABTS^+^• radical scavenging activity

ABTS^+^• free radical scavenging activity of LBPPs was determined by a previous method with minor modification [75]. Briefly, 100 μL LBPPs with various gradients (0.1–1 mg/mL) and 1 mL ABTS^+^• working solution were added to a 10 mL tube, mixed well and left at room temperature for 6 min. The absorbance values were measured at 734 nm using a microplate reader.

#### 2.10.3 Superoxide anion radical scavenging activity

The superoxide anion radical scavenging activity was evaluated by a modified method [76]. Different concentrations of LBPPs (0.1–1 mg/mL) were configured, and 50 μL was removed and added to a 96 well plate, followed by 50 μL NBT solution (156 μmol/L), 50 μL NADH solution (468 μmol/L) and 50 μL PMS solution (60 μmol/L), mixed well, and reacted in a water bath at 25 °C for 5 min, and the absorbance values were measured at 560 nm using a microplate reader.

### 2.11 In-situ monitoring of LBPPs dissolution and superoxide anion radical scavenging capacity

Except for extending the ultrasonic time to 40 min, the other parameters of the optimal ultrasound-assisted pulping parameters remained unchanged. During ultrasonic processing, 120 spectral information samples were collected (one sample every 20 s). Then the samples were centrifuged (10000 rpm/min for 10 min), and the supernatant was collected and stored at −4 °C, followed by the determination of LBPPs content and the superoxide anion radical scavenging capacity.

The partial least squares (Pls), interval partial least squares (Ipls), and synergy internal-pls (Si-Pls) were used to screen spectral intervals to reduce the impact of background and noise during the acquisition process, and the screened feature information was combined with LBPPs dissolution rate and superoxide anion radical scavenging capacity determined synchronously to establish a model.

### 2.12 Statistical analysis

All experiments were carried out in triplicate, and the data were expressed as the mean ± standard deviation. SPSS 26.0 software was used for one-way ANOVA, and *p* < 0.05 was consider to be statistically significant. The figures were plotted with Origin 2019b software, the near infrared spectrogram processing and model establishment were analyzed with Matlab R2020a software, and the physical field was simulated with COMSOL multiphysics 5.6.

## 3 Results and discussion

### 3.1 Optimization of LBP pulping assisted by slit dual-frequency ultrasound

**Fig. 2:**
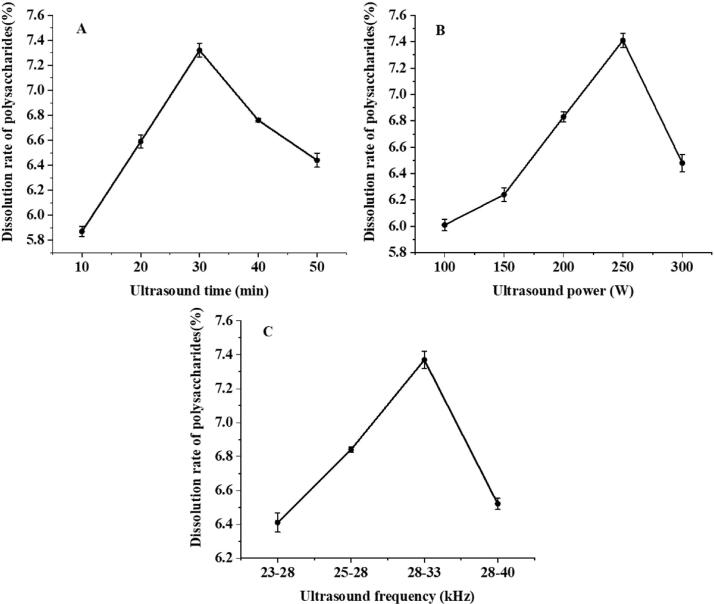
Effect of different treatment factors on the dissolution rate of LBPPs. Treatment time (A), Ultrasound power (B), Ultrasound frequency (C).

Single-factor experiments showed that the dissolution of LBPPs was significantly affected by ultrasonic time, power, and frequency modes. As shown in Fig. 2 (A), the LBPPs dissolution rate increased with time, it reached 7.32 % at 30 min, and then decreased rapidly, indicating that prolonged sonication time was conducive to the dissolution of LBPPs, but excessive ultrasonic treatment may destroy the glycosidic bond of polysaccharides, thereby reducing the dissolution rate. Similarly, in the range of 100–250 W, the dissolution rate of LBPPs increased and then began to decrease (Fig. 2B). This may be the result of enhanced cavitation, as moderate cavitation may facilitate solvents to enter the interior of LBPPs tissue and accelerate its dissolution, while excessive cavitation may lead to polysaccharide degradation and reduce dissolution rate. Studies have shown that dual-frequency ultrasound can directly destroy the cell wall of plant cells, contribute to the penetration of polysaccharides to the outside, and improve the effective ingredient dissolution rate [77]. The results of this study were consistent with previous reports (Fig. 2C). Based on these, the optimal level of each factor was preliminarily determined and then further optimized by response surface methodology.

The coefficient values of Eq. (2) were calculated and tested for their significance with Design Expert 10. The *p*-value was used as to check the significance of each coefficient. The smaller is the value of *p*, the more significant is the corresponding coefficient [78]. As shown in Table 2, the linear coefficients (*X_1_*, *X_2_*, *X_3_*), quadratic term coefficients (*X_1_^2^*, *X_2_^2^*, *X_3_^2^*) and cross product coefficients (*X_1_X_3_*, *X_2_X_3_*) were significant (*p* < 0.05), indicating that ultrasound power, ultrasonic frequency modes and time were all significantly correlated with the dissolution rate of LBPPs.

**Table 2:** Analysis of variance (ANOVA) for the fitted regression equation of dissolution rate.

Source	Sum ofsquares	df	Meansquare	*F*-value	*p*-value	Significant
Model	4.76	9	0.34	43.12	< 0.0001	**
*X_1_*	0.036	1	0.036	7.76	0.0146	*
*X_2_*	0.024	1	0.024	5.22	0.0384	*
*X_3_*	0.027	1	0.027	5.83	0.0301	*
*X_1_X_2_*	0.0077	1	0.008	1.66	0.2191	ns
*X_1_X_3_*	0.032	1	0.032	6.96	0.0195	*
*X_2_X_3_*	0.031	1	0.031	6.68	0.0216	*
*X_1_^2^*	1.16	1	1.16	48.31	< 0.0001	**
*X_2_^2^*	1.07	1	1.07	66.87	< 0.0001	**
*X_3_^2^*	1.73	1	1.73	73.77	< 0.0001	**
Residual	0.065	7	0.0047			
Lack of Fit	0.053	3	0.0053	1.82	0.2953	ns
Pure Error	0.012	4	0.003			
Cor Total	4.83	16				
*R^2^* = 0.9865	*R^2^_adj_*_=_0.9730	C.V.%=1.02%				

* Significant (*p*＜0.05); ** very significant (*p*＜0.01); ns: not significant (*p*＞0.05).

**Fig. 3:**
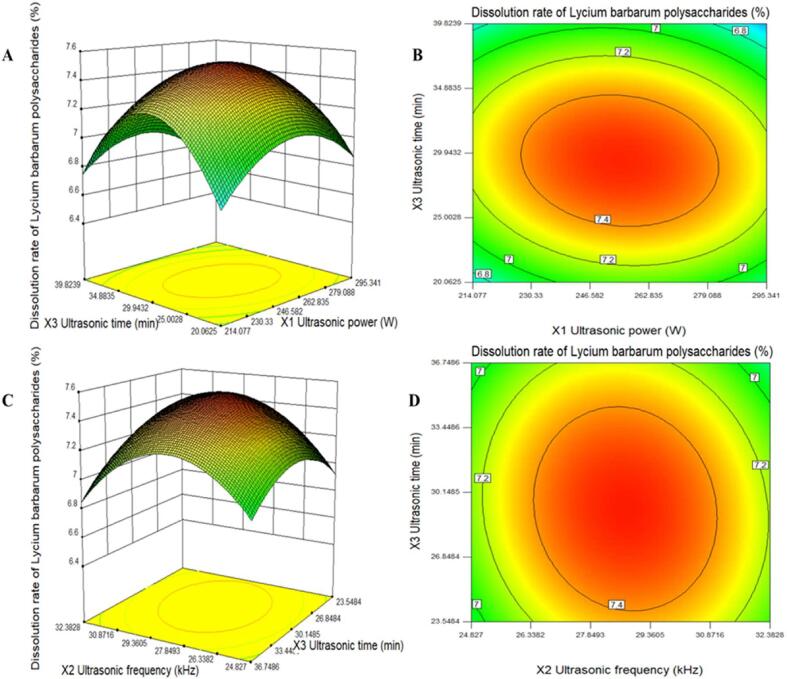
Response surface and contour plots of different factors. Ultrasonic power and ultrasonic time (A) and (B); Ultrasonic frequency and Ultrasonic time (C) and (D).

The response surfaces (3-D) and contour plots (2-D) were presented in Fig. 3. The shape of the contour plots represents the interaction between variables. Elliptical contour implies a perfect interaction between the independent variables, while circular contour is not the case [77]. As shown in Fig. 3 (A), the dissolution rate of LBPPs reached the maximum when the ultrasonic power reached 253.86 W, and then began to decline. Under certain ultrasonic power, when the ultrasonic time extended to 29.5 min, the dissolution rate of LBPPs increased rapidly and then began to decrease. Fig. 3 (B, D) shows that there is a significant interaction between different dual-frequency combinations and ultrasound time (*p*＜0.05).

In order to verify the optimal response value, the ultrasonic power, dual frequency combination and ultrasonic time were adjusted to 250 W, 28–33 kHz and 30 min. Under these conditions, the dissolution rate of LBPPs was 7.57 % ± 0.08, which was not significantly different from the predicted value (7.61 %, *p* > 0.05), indicating that the model was reliable and effective.

### 3.2 Effects of ultrasonic treatments on the components of LBP and LBPPs

Under optimal conditions of ultrasonic-assisted pulping, the main components in LBP and LBPPs were determined. As shown in Table 3, compared with NU-LBP, ultrasonic treatment not only significantly improved the dissolution of polysaccharides (increased by 84.6 %), but also increased the contents of total polysaccharides, reducing sugar, polyphenol, flavonoid, soluble protein, and carotenoid in LBP. Moreover, sonication remarkably decreased the molecular weight of LBPPs, and the contents of uronic acid and sulfate in LBPPs respectively increased by 22.5 % and 58.3 %, which play an important role in the biological activity of polysaccharides [24]. These results showed that ultrasonic treatment contributes to improve the dissolution of beneficial components.

**Table 3:** Effect of sonication on the components of LBP and LBPPs.

	NU-LBP	U-LBP	NU-LBPPs	U-LBPPs
Reducing-sugar(mg/g)	171.29 ± 0.47	215.96 ± 0.33	–	–
Polysaccharide(mg/g)	301.59 ± 0.12	556.73 ± 0.55	–	–
Polyphenol(mg/g)	1.48 ± 0.16	2.56 ± 0.21	–	–
Flavonoid(mg/g)	2.46 ± 0.28	3.53 ± 0.36	–	–
Soluble-Protein (mg/g)	0.06 ± 0.01	0.08 ± 0.01	–	–
Carotenoid(mg/g)	0.44 ± 0.06	0.54 ± 0.09	–	–
Total Polysaccharides(mg/g)	472.88	772.69	–	–
Sulfric-acid group (%)	–	–	0.12	0.19
Uronic acid (%)	–	–	36.78 ± 0.44	45.04 ± 0.59
*Mw* (g/mol)	–	–	4.467 × 10^5^	1.835 × 10^5^
*Mn* (g/mol)	–	–	4.166 × 10^5^	1.567 × 10^5^
*Mw*/*Mn*	–	–	1.072	1.171

Note: NU, No ultrasonication; U, ultrasonication.

### 3.3 Dissolution kinetics of polysaccharides in LBP

To better understand the dissolution behavior of polysaccharides in LBP, the dissolution rate of LBPPs was determined at various ultrasonic power and dual-frequency modes. Besides, the kinetic parameters, such as apparent rate constant *k*, relative raffinate rate, half-life, and effective diffusion coefficient were obtained, and the obtained kinetic models were fitted and verified, which are crucial for the optimization of LBP pulping process.

#### 3.3.1 Effect of sonication time, ultrasonic power and dual-frequency modes on LBPPs dissolution rate

**Fig. 4:**
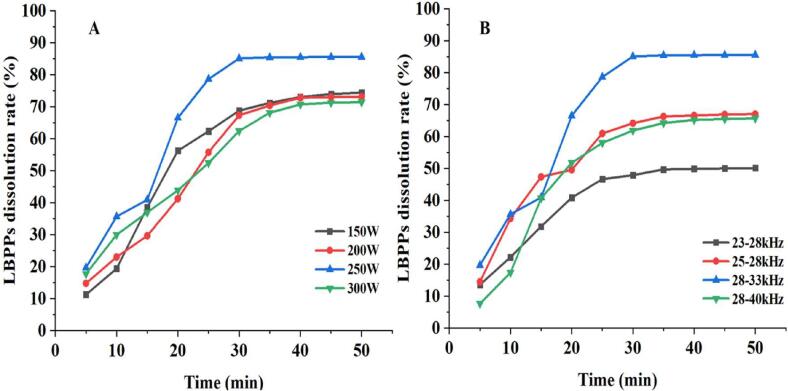
Relationship between sonication time and ultrasonic power (A) and dual-frequency combination (B).

Fig. 4 (A, B) shows that under fixed ultrasonic power and dual frequency mode, the dissolution rate of LBPPs can be increased by extending the ultrasonic time. When the ultrasonic time was 30 min, the dissolution rate of LBPPs was the highest at the ultrasonic conditions of 250 W and 28–33 kHz, which was much higher than other conditions, compared with 300 W and 28–40 kHz ultrasonic conditions, the dissolution rate of LBPPs increased by 20.9 % and 37.5 %, respectively. These results suggested that the effect of ultrasound treatment time on LBPPs dissolution rate is relatively limited, rather than the longer the better. Appropriate the ultrasonic power and frequency modes may be more effective in improving the dissolution rate of LBPPs, and the effect of the latter seems to be greater.

#### 3.3.2 Analysis of the rate constant *k*

**Fig. 5:**
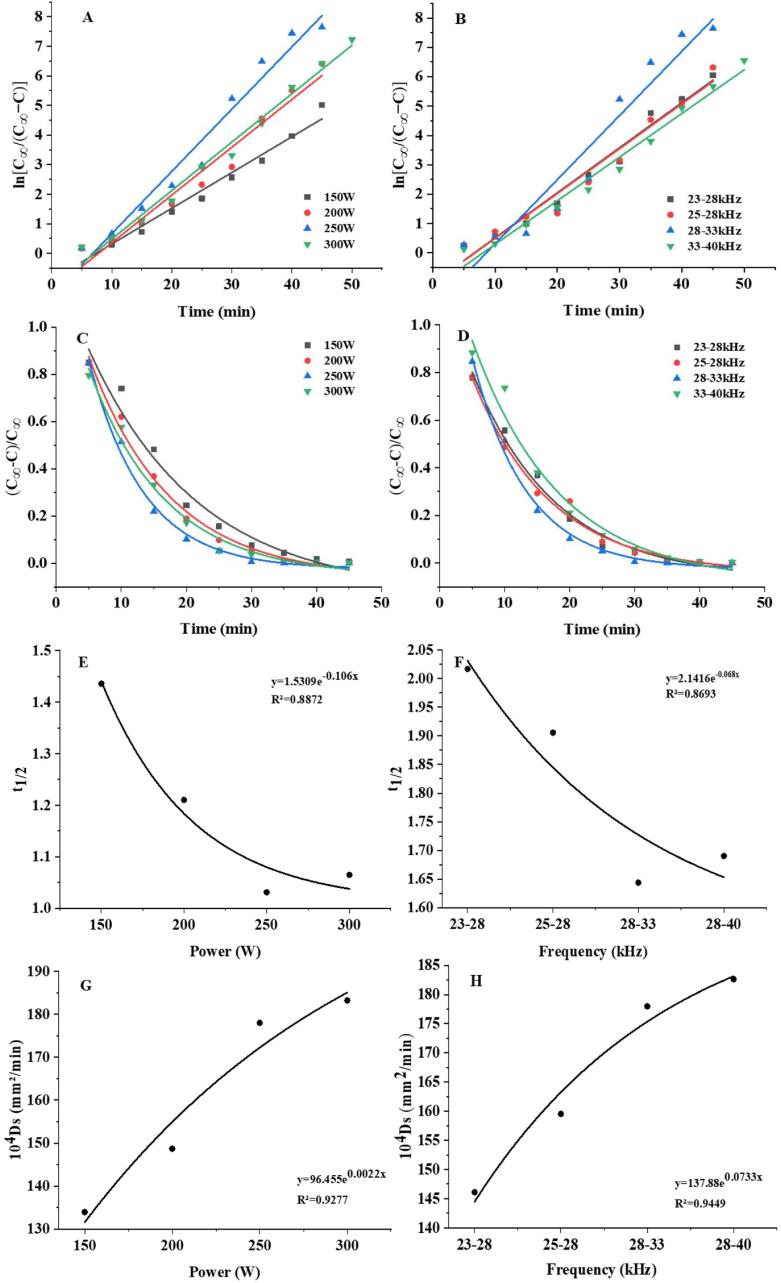
Kinetic correlation parameters of polysaccharide dissolution. Relationship between ln[C_∞_/(C_∞_−C)] and ultrasonic time under different ultrasonic powers (A) and dual-frequency combinations(B). Relationship between relative raffinate rate and ultrasonic time under different ultrasonic powers (C) and dual-frequency combinations (D). Relationship between t_1/2_ and ultrasonic power (E) and dual-frequency combinations (F). Relationship between Ds and ultrasonic power (G) and dual-frequency combinations (H).

The value of *k* represents the dissolution rate of LBPPs, and the larger the value, the faster the polysaccharides dissolve in LBP. As shown in Fig. 5 (A, B), the value of *Ln*[*C_∞_*/(*C_∞_*-*C*)] was linearly correlated with time under arbitrary ultrasonic power and dual-frequency combinations, and the correlation coefficients (*R_Power_* > 0.9087, *R_Frequency_* > 0.9335) indicated that the data fit well with the calculated values of kinetic model, which is suitable for predicting the dissolution rate of LBPPs during pulping (Table.4). Besides, the value of *k* at 250 W and 28–33 kHz were significantly higher than that of other power and frequency modes, which may be due to the fact that within a certain range, the increase in power intensity and frequency helps to break the cell wall of *Lycium barbarum*, accelerate the process of solvent penetration, as well as facilitate the diffusion of LBPPs from inside to outside [79], making it easier for the internal components to penetrate the cell wall and diffuse into the solvent, thereby promoting the dissolution of polysaccharide components.

**Table 4:** Dissolution kinetics related parameters of LBPPs.

Power/W	*k*	*b*	*R^2^*	Frequency/kHz	*k*	*b*	*R^2^*
Rate constant							
150	0.1361	−1.2357	0.9713	23–28	0.1545	−1.1996	0.9335
200	0.1615	−1.6145	0.9087	25–28	0.1635	−1.2235	0.9631
250	0.1896	−1.6698	0.9267	28–33	0.1896	−1.6698	0.9267
300	0.1836	−1.8311	0.9852	28–40	0.1844	−2.0376	0.9778

Relative raffinate rate							
150	0.1211	2.4694	0.9624	23–28	0.1541	2.7888	0.9633
200	0.1610	3.5106	0.9883	25–28	0.1584	3.0794	0.9656
250	0.2123	4.2058	0.9915	28–33	0.2171	4.2058	0.9761
300	0.1661	2.9542	0.9736	28–40	0.1433	3.0118	0.9474

#### 3.3.3 Analysis of relative raffinate rate

The relative raffinate rate *y*=(*C_∞_*-*C*)/*C_∞_* reflects the ratio of the undissolved active ingredient in the sample to the concentration of the active ingredient when dissolution reaches equilibrium. The fitting analysis was performed by taking *y* as the ordinate and ultrasound time *t* as the abscissa to obtain the fitting curve and specific parameters of the equation. As shown in Table 4, the relative raffinate rate was linearly related to sonication time (*R^2^* > 0.9), under any power and dual-frequency combination, the relative raffinate rate decreased with the extension of ultrasonic time, with the lowest at 250 W and 28–33 kHz, indicating that under these conditions, most LBPPs were thoroughly dissolved and the amount of undissolved was the least (Fig. 5 C, D). In addition, as the sonication time approached 30 min, the relative raffinate rate gradually decreased to the lowest and then remained unchanged, demonstrating that prolonged time is not conducive to the dissolution of polysaccharides.

#### 3.3.4 Analysis of half-life period (*t_1/2_*)

Half-life (*t_1/2_*) refers to the time taken for the concentration of the sample to decrease to one-half of its original value. As shown in Fig. 5 (E, F), at 250 W and 28–33 kHz, the value of *t*_*1/2*_ was the lowest, suggesting the increase in power and frequency accelerated the dissolution rate of LBPPs, which may be that the cavitation intensified the fragmentation of the granulosa cell wall, making it easier for polysaccharide components to dissolve. However, the values of *t*_*1/2*_ decreased at 300 W 28–40 kHz, despite the cavitation effect was further strengthened. Studies have showed that excessive energy intensity generates more bubbles through reflecting sound waves and then reduces energy transmission. Moreover, high-energy ultrasound may cause the degradation or isomerization of effective ingredients, thereby reducing the dissolution rate and concentration content of active ingredients [80].

#### 3.3.5 Analysis of diffusion coefficient (*Ds*)

The equilibrium concentration represents the limit of LBPPs diffusion and migration, while the diffusion coefficient reflects the speed of LBPPs migration. As shown in Fig. 5 (G, H), within a certain range, the higher the ultrasonic power/ultrasonic frequency was, and the faster the diffusion and migration. This may be related to that strong cavitation effect reduces material particles and promotes mass transfer, as the apparent rate constant *k* is negatively correlated with the radius (*R*) of *Lycium barbarum* particles (Eq.7). However, at 300 W and 28–40 kHz, the increase in diffusion velocity of LBPPs was not significant, and considering green energy conservation, the ultrasound conditions of 250 W and 28–33 kHz were finally chosen. The relationship between Ds and ultrasound power and dual-frequency can be described by the exponential models, which fit well with *Ds* (*R_Power_* > 0.9277, *R_Frequency_* > 0.9449), suggesting that the diffusion coefficient can be obtained according to them.

### 3.4 Physical field coupling in ultrasonic processes

#### 3.4.1 Simulation of sound field

**Fig. 6.1:**
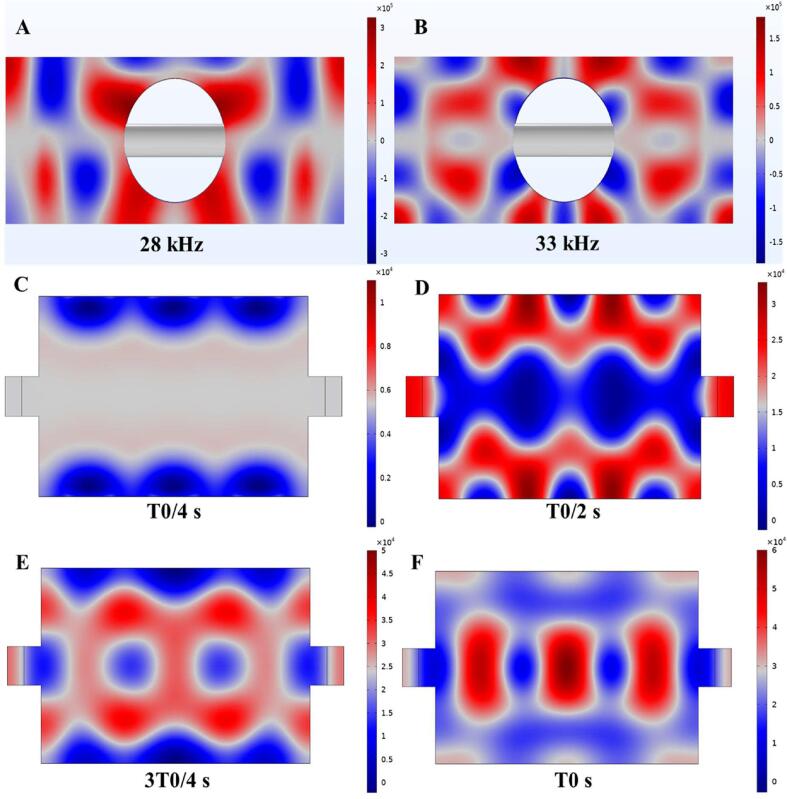
Absolute sound pressure (Pa) distribution under different ultrasound frequency modes. Single frequency ultrasound mode (A, B). Dual frequency ultrasound mode (C - F).

The magnitude and amplitude distribution of sound pressure at different position in a narrow cavity can reflect the intensity and uniformity of the sound field. As shown in Fig. 6.1, at 28 kHz and 33 kHz, there is a large area of high sound pressure area at the wave belly of the cavity, the sound field distribution remains relatively uniform, which is more conducive to reaching the cavitation threshold and generating cavitation effect [81], which has a positive effect on improving the quality and efficiency of the target products.

The sound field distribution of dual frequency ultrasound was studied at T0/4 s,T0/2 s, 3 T0/4 s, T0 s. Under 28–33 kHz (Fig. 6.1, C-F), the sound wave was emitted from the transducer position and then diffused around. Superposition of two train waves with different frequencies can greatly weaken the generation of standing wave effect and improve the efficiency of sonochemical reaction [82]. Compared with single frequency ultrasound, the reduction of standing wave area of dual frequency ultrasound can effectively improve the uniformity of sound field.

#### 3.4.2 Simulation of flow field

**Fig. 6.2:**
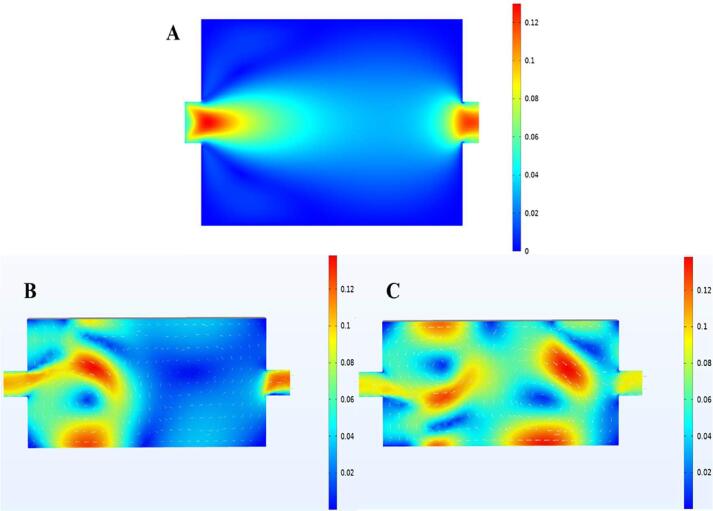
Flow field distribution under different conditions. No ultrasonic excitation (A). Single frequency ultrasonic excitation (B). Dual frequency ultrasonic excitation (C).

The flow conditions of natural circulation (laminar flow) were simulated without ultrasonic excitation (Fig. 6.2A), and the overall flow was gentle. Due to the restriction of narrow area, the inlet and outlet flow rates were higher than those in other locations. The flow field distribution under ultrasonic excitation was shown in the Fig. 6.2 (B, C), and the overall velocity of flow field was significantly improved. Under ultrasonic excitation, the flow field was obviously disturbed, in the form of a jet formed along the direction of sound wave propagation [83]. Under dual frequency ultrasonic excitation, with the superposition of different sound waves, more complex vibration will be generated, resulting in more obvious sound flow [84], which was confirmed by the eddy current generated in the simulation results.

#### 3.4.3 Simulation of temperature field

**Fig. 6.3:**
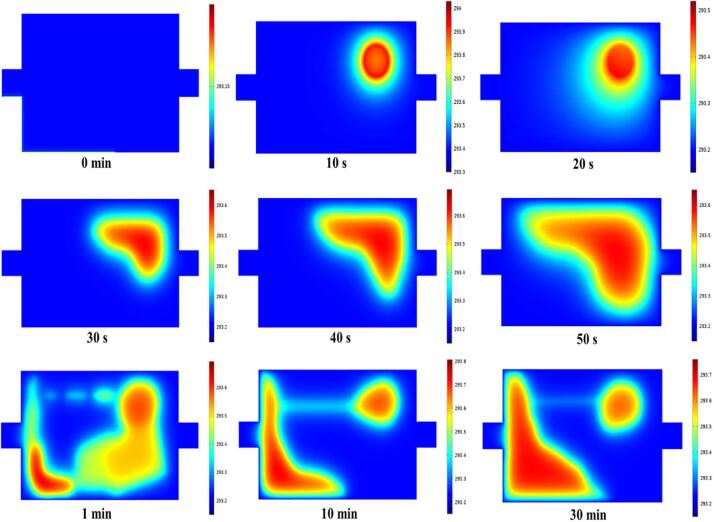
Temperature (K) field distribution under ultrasonic excitation for 0–30 min.

The initial temperature was room temperature (293.15 K), and the ultrasonic frequency was 28 kHz. Using laminar flow as the flow background, simulate the temperature change at 0–30 min (Fig. 6.3). The ultrasonic heat source was generated in the center of the transducer, and the maximum temperature was 294 K. In the first 30 s, the heat generated by ultrasonic wave gradually transferred to the other positions of the cavity with fluid flow. Between 30 s and 30 min, the maximum temperature transfered to the wall near the cavity inlet, and the maximum temperature was 293.8 K. During the treatment time of 30 min, the temperature in the slit ultrasonic reaction chamber rises < 1 K, and does not increase after reaching the maximum temperature. During fluid flow process, heat accumulation occurs locally, but the generated heat does not seem to have a significant impact on the overall temperature change, which is similar with previous studies [85]. In addition, the thermal effect of ultrasound is closely related to the ultrasonic sound intensity [86], compared with probe type ultrasound, slit ultrasound generates much less sound intensity, thus the heat generation and its impact on the temperature in the treatment chamber are relatively small.

### 3.5 Structural characteristics of polysaccharides in LBP

#### 3.5.1 UV–Vis spectroscopy

**Fig. 7:**
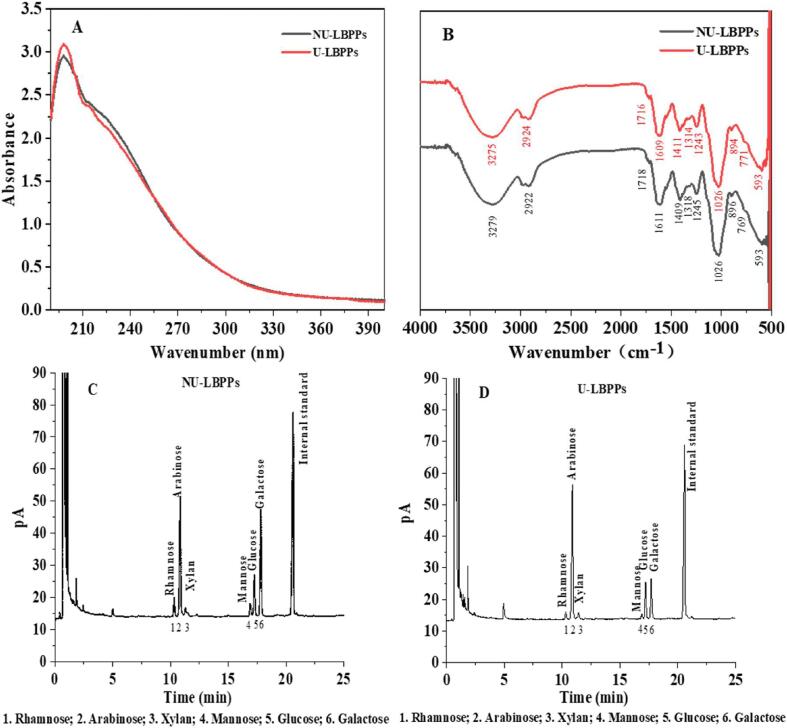

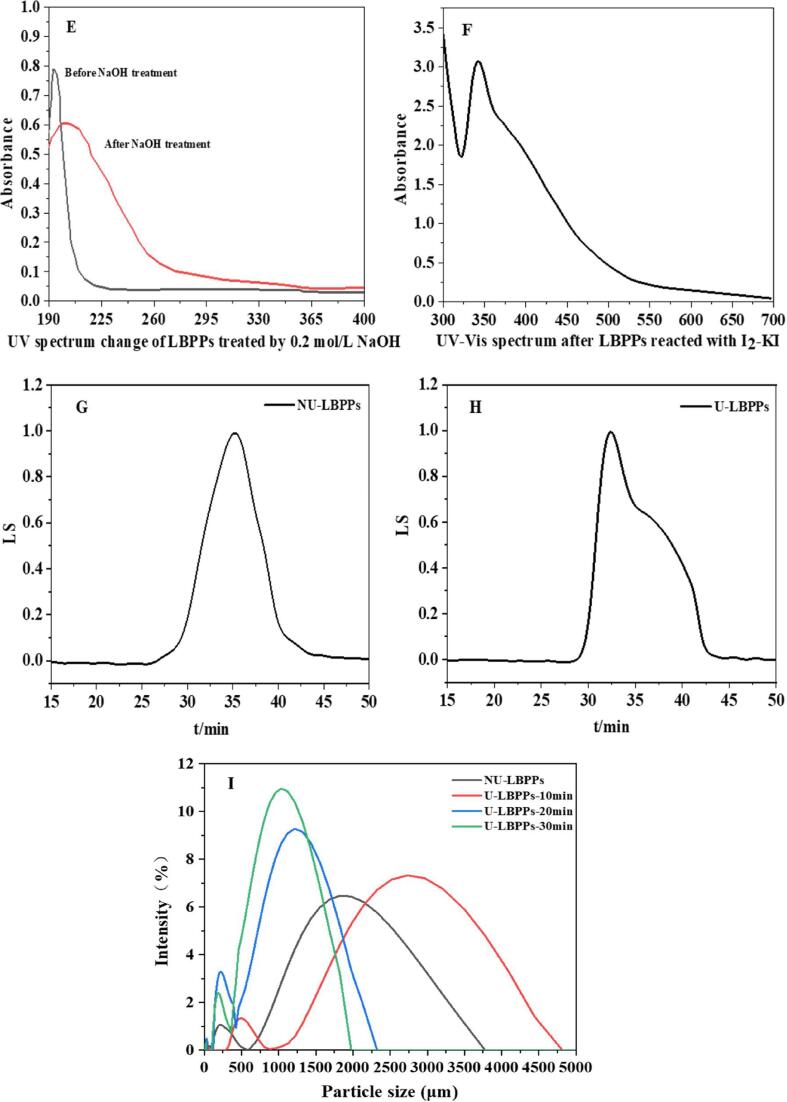
Structural Analysis of LBPPs. UV–Vis spectrogram (A), FT-IR spectrogram (B), Monosaccharide composition of NU-LBPPs (C), Monosaccharide composition of U-LBPPs (D), β-Elimination reaction (E), I-KI reaction (F), Molecular weight of NU-LBPPs (G), Molecular weight of U-LBPPs (H), Structural Analysis of LBPPs. Particle size (I).

As shown in Fig. 7 (A), both LBPPs showed weak absorption peak at 280 nm, suggesting that although free proteins had been cleared by Sevag reagent, there may still be a small amount of protein in the form of glycoproteins [24]. In addition, no absorption peak was found at 260 nm, indicating that LBPPs was free of nucleic acids [87].

#### 3.5.2 FT-IR spectra characteristics of LBPPs

FT-IR spectra were used to analyze the type and vibration of functional groups in LBPPs. As shown in Fig. 7 (B), LBPPs showed wide and strong absorption peaks at 3279 cm^−1^ (NU-LBPPs) and 3275 cm^−1^ (U-LBPPs), which are the stretching vibration of hydroxyl group on the free hydrogen bond of polysaccharides; The absorption peaks at 2922 cm^−1^ (NU-LBPPs) and 2924 cm^−1^ (U-LBPPs) caused by the vibration of methylene C-H, which are the characteristic absorption peaks of polysaccharides (Li et al., 2019); The absorption peaks at 1718 cm^−1^ (NU-LBPPs) and 1716 cm^−1^ (U-LBPPs) are H based C = O stretching vibration [88], indicating that LBPPs contains uronic acid, which is consistent with the GC determination results. The absorption peaks at 1611 cm^−1^ (NU-LBPPs) and 1609 cm^−1^ (U-LBPPs) are the crystal water of polysaccharides or variable angle vibration of primary amino acid N–H, indicating that crystal water or bound protein may be present in the polysaccharides. In addition, the variable angle vibration peak of O-H in hydroxyl at 1409 cm^−1^ (U-LBPPs) and 1411 cm^−1^ (U-LBPPs) suggested that LBPPs contains carboxyl groups. The absorption peak near 1245 cm^−1^ (NU-LBPPs) and 1243 cm^−1^ (U-LBPPs) are the stretching vibration of pyranose ring. The absorption peaks at 894 cm^−1^ (NU-LBPPs) and 896 cm^−1^ (U-LBPPs) are the ring vibration absorption peaks of sugar, revealing that the glycosidic bond of LBPPs is mainly *β*-configuration. FT-IR results showed that ultrasonic treatment did not change the important functional groups in LBPPs.

#### 3.5.3 Monosaccharide composition of LBPPs

Monosaccharide composition is closely related to the structure and biological activity, which was analyzed by GC. As shown in Fig. 7 (C, D), the monosaccharides in NU-LBPPs are rhamnose, arabinose, xylan, mannose, glucose and galactose, with the contents of 11.53 %, 26.48 %, 9.26 %, 12.05 %, 16.87 % and 23.81 %. The monosaccharides in U-LBPPs are rhamnose, arabinose, xylan, mannose, glucose and galactose, and the contents are 3.10 %, 50.27 %, 5.45 %, 10.11 %, 14.10 % and 16.97 %, respectively. These results indicated that ultrasonic treatment did not change the composition of monosaccharides in LBPPs, but changed their molar percentage, which was consistent with previous reports [89].

Notably, after ultrasonic treatment, the molar ratio of arabinose increased from 26.48 % to 50.27 %, while the molar ratio of other monosaccharides decreased to a certain extent. This may be that ultrasonic treatment changed the arrangement of hydrogen atoms and hydroxyl groups on carbon atoms [90], thus transforming the other monosaccharides into arabinose.

#### 3.5.4 Glycopeptide linkage types and side chain branching

**Fig. 8:**
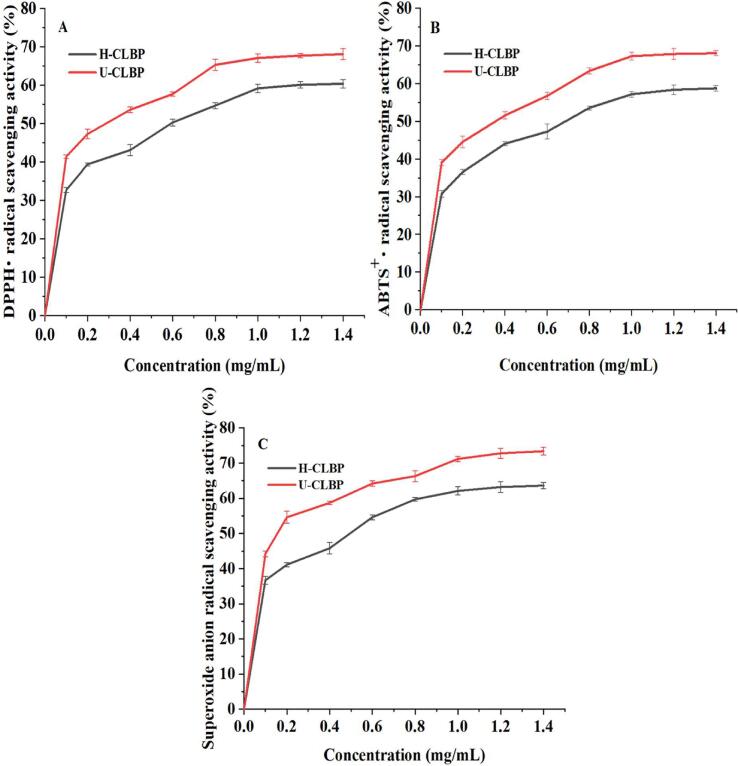
Antioxidant activity of LBPPs. DPPH• (A), ABTS^+^• (B) and Superoxide anion radical (C).

*β*-Elimination reaction is a simple and rapid method to detect the glycopeptide bond. The O-glycopeptide linkage is extremely unstable under alkaline conditions, and serine and threonine linked to the glycopeptide bond will be converted to α-amino-acrylic acid and α-amino-butenoic acid [91], [92]. Both of them have obvious absorption values at 240 nm, which is confirmed in Fig. 8 (E), indicating that LBPPs contain O-glycopeptide linkage. Similarly, as shown in Fig. 8 (F), the reactant of LBPPs and I_2_-KI had a maximum absorption at 350 nm, and no maximum absorption occured at 565 nm [93], indicating that LBPPs may have longer side chains and more branches, which may be one of the reasons for LBPPs strong antioxidant activity [94].

#### 3.5.5 Molecular weight of LBPPs

The molecular weight of polysaccharides is closely related to their biological activities, which are greatly affected by the treatment methods [95]. As shown in Fig. 8 (G, H), NU-LBPPs exhibited a single symmetrical molecular weight distribution, indicating that the polysaccharides are highly homogeneous. However, U-LBPPs appeared a distinct shoulder peak, suggesting that the molecular weight of polysaccharides changed from uniform to non-uniform. In addition, ultrasonic treatment significantly decreased the molecular weight (*Mw*) of LBPPs (Table 3), which may be due to the breakage of LBPPs glycoside chain caused by ultrasound cavitation, leading to the reduction of the *Mw*. Polydispersity coefficient (*Mw*/*Mn*) represents the molecular weight dispersion degree of polysaccharides. Smaller *Mw*/*Mn* indicates a more uniform molecular weight distribution [96]. The polydispersity coefficient (*Mw*/*Mn*) of U-LBPPs increased, indicating that its molecular weight changed from homogeneous to uneven, which is consistent with the distribution of chromatographic peaks shown in Fig. 8 (H).

#### 3.5.6 Particle size distribution

The particle size can indirectly reflect the aggregation and solution behavior of polysaccharide molecules [30]. The smaller the distance between particles, the more concentrated the distribution of particles, and the higher the degree of polysaccharides size homogenization. As shown in Fig. 9 (I), the particle size of LBPPs decreased with the increase of ultrasonic time, indicating that the particle size distribution was gradually concentrated. This may be due to the weakening of hydrogen bonding interactions between polysaccharide molecules and the fragmentation of polysaccharide chains caused by ultrasound, which not only reduced the diffusion distance of compounds in the matrix, but also increased the contact area between the matrix and the solvent, thus promoting the dissolution of polysaccharide components in *Lycium barbarum*. The appearance of heterozygous peaks may be related to the interweaving and binding of polysaccharide molecular chains, resulting in uneven particle size distribution. Studies have shown that high molecular weight polysaccharides tend to have larger particle size, which could explain the larger particle size of NU-LBPPs.

### 3.6 Antioxidant activity

The molecular weight of polysaccharides is closely associated with their antioxidant activity [97]. As shown in Fig. 8, the antioxidant activity of U-LBPPs was obviously higher than that of NU-LBPPs (*p* < 0.05). It has been confirmed that β-glycoside bonds make polysaccharides more capable of scavenging free radicals [98], and the presence of natural antioxidant α-aminobutyric acid also greatly improves the antioxidant capacity of LBPPs. The antioxidant activity of LBPPs increased after sonication, which may be due to the fact that ultrasonic treatment not only disrupted the natural hydrogen bonds of polysaccharides, causing more hydrogen atoms to be transferred to neutralize free radicals [99], but also altered the composition of monosaccharides and promoted the dissolution of antioxidant components. In addition, the content of uronic acids in polysaccharides significantly increased after ultrasonic treatment, which may be the main reason for the improvement of antioxidant activity of LBPPs. Studies indicated that particle size had a significant effect on antioxidant activity [100], and the increased antioxidant activity of U-LBPPs was closely related to the improved uniformity of dissolved polysaccharides and reduced particle size. Furthermore, sonication reduced the molecular weight of polysaccharides [101] enhanced the inhibition ability of polysaccharides on free radicals, which may be related to that low molecular weight polysaccharides are more vulnerable to the attack of free radicals [102]. Fig. 8 (C) showed that superoxide anion radicals were the most sensitive to LBPPs and had the highest scavenging ability. Therefore, superoxide anion radical was selected as the object to build the prediction model of NIR spectrum.

### 3.7 Correlation analysis

In order to evaluate the effect of ultrasonic treatment on LBPPs dissolution during the pulping process, the correlation between sonication and LBPPs content, particle size and antioxidant activity was analyzed. The higher the absolute value of the correlation coefficient, the closer the correlation between them. As shown in Table 5, ultrasonic treatment significantly correlated with the content of LBPPs (*p* < 0.01), particle size and scavenging ability against superoxide anion radicals (*p* < 0.05). No significant correlation was found between sonication and the ability to scavenge DPPH• and ABTS^+^• (*p* > 0.05). These results showed that sonication has a significant positive effect on LBPPs content, particle size uniformity and scavenging ability against superoxide anion radicals. In view of the close relationship between ultrasonic treatment and polysaccharide content and superoxide anion radical scavenging rate, it can be used as an important parameter to establish a real-time monitoring model.

**Table 5:** Correlation analysis between ultrasonication and LBPPs content in LBP, particle size and antioxidant activity.

	LBPPs content	Particle size	DPPH•scavenging	ABTS^+^•scavenging	Superoxide anionscavenging
*r*					
U-T	0.721**	0.546*	0.426	0.481	0.690*
*p*					
U-T	0.006	0.043	0.474	0.402	0.039

Note: Pearson correlation coefficient ® and significance level (P-value);

U-T, ultrasonic treatment;

* Significant correlation at the 0.05 level (two-tailed);

** Significant correlation at the 0.01 level (two-tailed).

### 3.8 Establishment of in situ real-time monitoring model

#### 3.8.1 Pretreatment of original spectrum

**Fig. 9.1:**
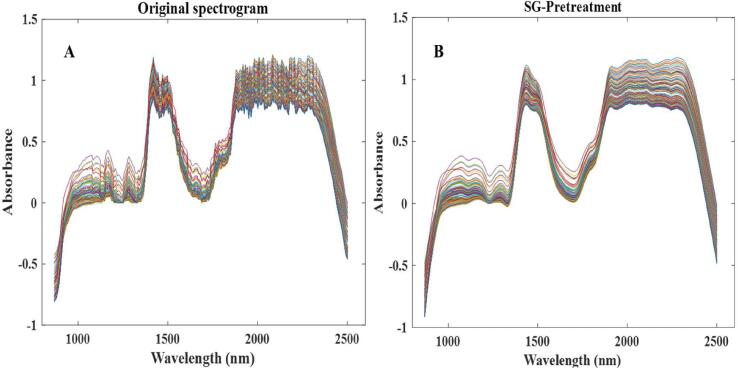
Original spectrogram (A) and SG-Pretreatment spectrogram (B).

NIR combined with off-line sampling was used to detect LBPPs content and superoxide anion radical scavenging capacity during pulping process, and an in situ real-time monitoring model for ultrasonic assisted fresh fruit pulping of *Lycium barbarum* was established. The original NIR spectrum in the process of pulping was shown in Fig. 9.1 (A), it was interfered by some information unrelated to the changes in LBPPs content and antioxidant activity [103]. Therefore, the original spectrograms need to be preprocessed by stoichiometric methods to eliminate interference and useless information before model calibration. The common preprocessing methods include Centre, Multiple scattering correction (MSC), Sgolayfilt (SG), Standard normal variate transform (SNV), First derivative (1st), Second derivative (2nd) and their combinations, respectively. Based on these methods, the Pls model was established, and the correction model was used to predict the LBPPs dissolution rate and superoxide anion radical scavenging capacity of LBP (Table 6). Among them, the correlation coefficient (*R^2^*), root mean squared error of cross-validation (*RMSECV*), root mean squared error of prediction (*RMSEP*) were used as evaluation indexes to screen the optimal pretreatment methods. The smaller the values of *RMSECV* and *RMSEP*, the stronger the prediction ability of the model for unknown samples. The higher the *R*, the closer to 1, indicating the higher the correlation between the measured value and the predicted value. As shown in Table 6, the SG algorithm had a clear concept and simple calculation. By properly selecting relevant parameters, it can effectively suppress the interference of high-frequency component signals, and obtain low-frequency component signals containing necessary information. Therefore, it can be effectively applied to the denoising and smoothing of spectral data. In addition, the SG algorithm also causes time shift, which can reflect the signal shift before and after denoising, helping to accurately solve the real-time variation parameters of polysaccharide content in samples. This method was simpler and faster than the others, and retains the distribution characteristics of relative maximum, minimum and width. After comparison, SG was considered the most effective pretreatment method. Fig. 9.1 (B) showed the powerful “impurity removal” effect of SG. Consequently, the spectra processed by SG were selected for the following modeling analysis.

**Table 6:** Comparison of different pretreatment methods.

Pretreatment method	Major component	Results of Pls analysis			
		*Rc*	*RMSECV*	*Rp*	*RMSEP*
Polysaccharide dissolution rate					
MSC	6	0.9625	0.1350	0.9719	0.0529
MSC and 1st	10	0.9543	0.1370	0.9622	0.0615
SNV	9	0.9634	0.1338	0.9740	0.0511
SNV and 1st	10	0.9577	0.1308	0.9644	0.0605
SG	8	0.9807	0.0977	0.9820	0.0459
SG and 1st	8	0.9592	0.1410	0.9672	0.0582
Center	8	0.9635	0.1332	0.9775	0.0478
Center and 1st	6	0.9518	0.1403	0.9644	0.0616
Normalize	9	0.9699	0.1338	0.9759	0.0511
Normalize-1st	10	0.9615	0.1290	0.9558	0.0682
1st	4	0.9483	0.1473	0.9520	0.0734
2nd	3	0.8695	0.2000	0.8840	0.1064

Radical scavenging capacity					
MSC	7	0.9747	0.1792	0.9804	0.0794
MSC and 1st	6	0.9688	0.1592	0.9801	0.0782
SNV	7	0.9798	0.1592	0.9824	0.0758
SNV and 1st	5	0.9715	0.1828	0.9778	0.0824
SG	6	0.9852	0.1357	0.9855	0.0670
SG and 1st	5	0.9756	0.1828	0.9742	0.0915
Center	8	0.9765	0.1719	0.9797	0.0818
Center and 1st	4	0.9708	0.1846	0.9791	0.0822
Normalize	7	0.9821	0.1379	0.9847	0.0725
Normalize-1st	4	0.9716	0.1849	0.9732	0.0927
1st	4	0.9721	0.1820	0.9723	0.0966
2nd	4	0.8510	0.3718	0.8836	0.1888

Pls, Ipls and Si-Pls were used to establish a quantitative model to predict LBPPs dissolution rate and superoxide anion radical scavenging capacity during ultrasound-assisted fresh fruit pulping of *Lycium barbarum*. The stability and accuracy of the model were analyzed by comparing *Rc*, *Rp*, *RMSECV*, and *RMSEP* values.

#### 3.8.2 In-situ monitoring and analysis of polysaccharide dissolution rate

Although the spectra have been preprocessed, the impact of interference information cannot be completely eliminated. In addition, the signal information in certain intervals of the spectrum is weak and lacks correlation with samples, which leads to increased computational workload and decreased model accuracy. Therefore, it is necessary to further optimize the data through multivariate correction models, including filtering out the feature data, refining the data volume [104], removing the interference of irrelevant information, and thereby improving the precision and predictive ability of the model.

**Fig. 9.2:**
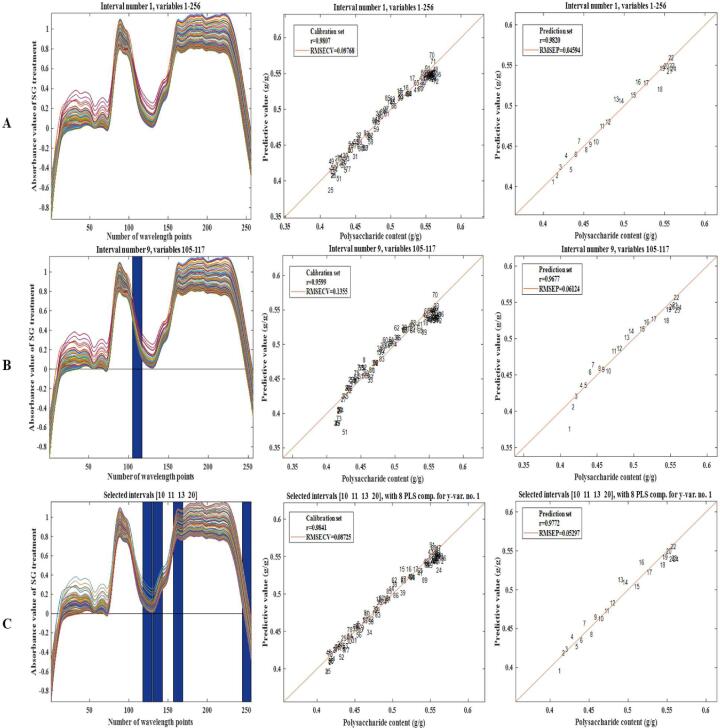
In-situ monitoring of polysaccharide dissolution rate in LBP sample. Calibration set and prediction set of Pls model (A), Calibration set and prediction set of Ipls model (B), Calibration set and prediction set of Si-Pls model (C).

Pls, Ipls and Si-Pls were used to optimize the quantitative prediction model for the polysaccharide dissolution and antioxidant activity. Fig. 9.2. showed the modeling effect of three multivariate corrected models. For Si-Pls, when the 20 sub intervals were divided and 4 sub intervals were combined (the 10th, 11th, 13th, and 20th), the corresponding spectral intervals were 1625.39 ∼ 1702.19 nm, 1708.59 ∼ 1785.24 nm, 1874.51 ∼ 1950.88 nm, and 2431.81 ∼ 2501.06 nm respectively. Four sub-intervals only accounted for 18.32 % of the entire spectral data, eliminating the interference of redundant data on model accuracy and calculation speed. The spectral band change in the range of 1874.51 ∼ 1950.88 nm may be caused by O-H stretching and O-H deformation mode of R-OH group of glucose and fructose, while the spectral band change in the range of 2431.81 ∼ 2501.06 nm may be caused by C-H stretching and C-H deformation mode of polymer formed by Maillard reaction. Consequently, Maillard reaction could be the result of the change of hydrocarbons related spectral bands. Table 7 showed that, the polysaccharide dissolution rate models established by Pls, Ipls, and Si-Pls, all had good predictive performance and achieved rapid detection of polysaccharides dissolution rate, Si-Pls exhibited the best predictive effect on the dissolution rate of LBPPs compared with Pls and Ipls.

**Table 7:** Comparison of different prediction models.

Model	Major component	Model analysis results			
		*Rc*	*RMSECV*	*Rp*	*RMSEP*
Polysaccharide dissolution rate					
Pls	8	0.9807	0.0977	0.9820	0.0459
Ipls	4	0.9599	0.1355	0.9677	0.0612
Si-Pls	8	0.9841	0.0873	0.9772	0.0530

Radical scavenging capacity					
Pls	6	0.9852	0.1357	0.9855	0.0670
Ipls	3	0.9827	0.1460	0.9802	0.0808
Si-Pls	9	0.9874	0.1246	0.9868	0.0665

#### 3.8.3 In-situ monitoring and analysis of superoxide anion radical scavenging capacity

**Fig. 9.3:**
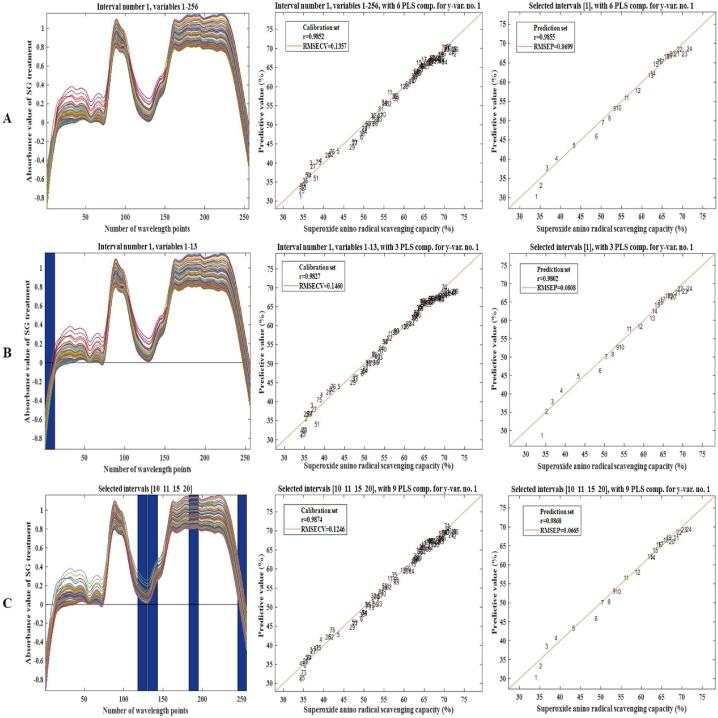
In-situ monitoring of superoxide radical scavenging capacity in LBP sample, Calibration set and prediction set of Pls model (A), Calibration set and prediction set of Ipls model (B), Calibration set and prediction set of Si-Pls model (C).

As shown in Fig. 9.3 (C), among the models for real-time monitoring of superoxide anion radical scavenging ability, Si-Pls performed best in terms of accuracy and effectiveness. In 20 sub intervals, the [10], [11], [15], [20] corresponded to 1625.39 ∼ 1702.19 nm, 1708.59 ∼ 1785.24 nm, 2039.82 ∼ 2115.92 nm and 2431.81 ∼ 2501.06 nm, respectively. Four sub-intervals only accounted for 18.30% of whole spectral data, the interference of redundant data on the model was eliminated, and the accuracy and calculation speed of the model was improved. Table 7 indicated that Si-Pls model had the highest calibration and prediction accuracy, which could better predict the real-time change of superoxide anion radical scavenging ability.

## 4 Conclusion

Ultrasonic assisted pulping of fresh *Lycium barbarum* fruit is an effective way to improve the processing efficiency and economic benefits of LBP. Under appropriate ultrasound conditions, the content of polysaccharides and other active ingredients in LBP can be significantly improved. Ultrasonic treatment can significantly change the proportion of monosaccharides in LBPPs, decrease the relative molecular weight and particle size, but increase the homogeneity of polysaccharides, which are closely related to the improvement of antioxidant activity. In addition, through the establishment of the dynamic model of polysaccharide dissolution in ultrasonic assisted pulping process and the visual simulation of multi physical fields (sound field, flow field, temperature field) were established to further clarify the dissolution law of polysaccharide and the micro mechanism of ultrasonic effect. Based on this, a real-time monitoring model of ultrasound assisted fresh fruit pulping process of *Lycium barbarum* was established to improve quality control efficiency and active strength assurance of the pulping process. These findings indicated that slit dual-frequency ultrasound has great potential in improve the quality of *Lycium barbarum* pulp, which may provide a theoretical support for the industrial development of smart systems for polysaccharides preparation. The real-time monitoring of NIR spectroscopy shows good performance and prediction accuracy, but it is still difficult to apply in large-scale food industry production, which needs to ensure the stability of the monitoring environment and model operation. Therefore, it is necessary to develop more stable and better prediction accuracy model algorithm to be applied to the food industry in the future.

## CRediT authorship contribution statement

**Tianyu Kong:** Investigation, Writing – original draft. **Shuhan Liu:** Investigation, Validation. **Yuqin Feng:** Methodology, Validation. **Yanli Fan:** Investigation, Validation. **Junwei Yu:** Investigation, Validation. **Haihui Zhang:** Writing – review & editing, Funding acquisition. **Meihong Cai:** Investigation, Validation. **Haile Ma:** Investigation, Validation. **Yuqing Duan:** Supervision, Project administration.

## Declaration of Competing Interest

The authors declare that they have no known competing financial interests or personal relationships that could have appeared to influence the work reported in this paper.
